# Labetalol, Nifedipine, and Methyldopa for the Management of Hypertensive Disorders in Pregnancy in Iran: A Cost‐Effectiveness Analysis

**DOI:** 10.1002/hsr2.72968

**Published:** 2026-08-09

**Authors:** Zahra Goudarzi, Mojtaba Nouhi, Najmeh Bordbar

**Affiliations:** ^1^ Health Human Resources Research Center Shiraz University of Medical Sciences Shiraz Iran; ^2^ Health Economics Department, School of Medicine Shahed University Tehran Iran; ^3^ Health Human Resources Research Center, School of Health Management and Information Sciences Shiraz University of Medical Sciences Shiraz Iran

**Keywords:** cost‐effectiveness, decision modeling, labetalol, methyldopa, nifedipine, pregnancy

## Abstract

**Background and Aims:**

Hypertensive disorders in pregnancy create major clinical and economic burdens on maternal health services, making evidence on efficient resource allocation and cost‐effectiveness essential for treatment decisions. However, no clear consensus exists on the most cost‐effective antihypertensive therapy in Iran. This study evaluated the cost‐effectiveness of methyldopa, labetalol, and nifedipine in pregnant women in Iran.

**Methods:**

A decision‐analytic model was created using a decision tree framework to compare the cost‐effectiveness of labetalol with nifedipine and methyldopa. The model used data from published randomized clinical trials and relevant literature. A hypothetical group of pregnant women aged 18 and older, at least 28 weeks pregnant, and experiencing severe hypertension (systolic blood pressure of 160 mmHg or higher, or diastolic blood pressure of 110 mmHg or higher), was studied over 4 months. The analysis considered the viewpoint of healthcare payers. Costs were measured in US dollars, and health outcomes were represented in quality‐adjusted life years (QALYs). A willingness‐to‐pay threshold of $18,261 per QALY was set. Deterministic and probabilistic sensitivity analyses, including 10,000 Monte Carlo simulations, were conducted to evaluate uncertainty.

**Results:**

In the base‐case analysis, nifedipine provided the highest QALYs (0.21), followed by labetalol (0.19) and methyldopa (0.16). The total costs were $607 for nifedipine, $500 for labetalol, and $847 for methyldopa. Labetalol was less expensive and more effective than methyldopa, making it the dominant choice. Compared to nifedipine, labetalol was not cost‐effective at the specified willingness‐to‐pay threshold. The probabilistic sensitivity analysis revealed that labetalol was cost‐effective in 70% of simulations against methyldopa and 29% against nifedipine.

**Conclusion:**

From the perspective of Iranian healthcare payers, labetalol is a cost‐saving and dominant option compared to methyldopa. However, it is not cost‐effective when compared to nifedipine at a willingness‐to‐pay threshold of $18,261 per QALY in managing hypertensive disorders during pregnancy.

## 1 Introduction

Hypertensive Disorders in Pregnancy (HDP) impact 10% of expectant mothers and account for 14% of maternal deaths across the world, ranking second only to obstetric hemorrhage [1, 2]. The occurrence of hypertension in primigravida and multigravida is 10%–15% and 2%–5%, respectively [3]. HDP contributes significantly to maternal mortality in various regions, accounting for 7% of pregnancy‐related deaths in the United States (US) [4]. Additionally, Latin America and the Caribbean report 25% of maternal deaths linked to HDP, developed countries at 16%, Asia at 9%, and Africa at 9% [5]. In Iran, HDP accounts for 17.1% of maternal deaths after hemorrhage [6].

High blood pressure during pregnancy is identified when the systolic BP (SBP) is equal to or greater than 140 mmHg and the diastolic BP (DBP) is equal to or greater than 90 mmHg, whether measured in a medical office or hospital setting. It is crucial to verify this diagnosis, ideally on two different occasions or at least 15 min apart in cases of severe hypertension [7]. HDP significantly contributes to maternal, fetal, and neonatal morbidity and mortality [3, 8]. Women who experience hypertensive disorders during pregnancy face an increased likelihood of developing various cardiovascular risk factors, including hypertension, type 2 diabetes, obesity, chronic kidney disease, premature cardiovascular disease (such as cardiac, cerebrovascular, and peripheral arterial conditions), and cardiovascular‐related mortality [9]. Hypertension that persists and is severe is regarded as a risk factor for maternal complications such as stroke, intracranial hemorrhage, cardiopulmonary decompensation, preeclampsia, and HELLP syndrome [3, 10]. The fetal risk includes intrauterine growth retardation, prematurity, intrauterine death, preterm birth with low birth weight, prolonged high‐level neonatal care, and postnatal death [8], as well as fetal decompensation resulting from decreased uterine blood flow, abruption, and stillbirth [2, 10]. Bellamy et al. conducted a meta‐analysis study that revealed that women with a history of preeclampsia are at a significantly higher risk of developing ischemic heart disease, stroke, and venous thromboembolic events. The risk is approximately doubled compared to women without such a history [11]. These findings highlight the importance of monitoring and managing the health of women who have experienced hypertensive disorders during pregnancy to ensure the safety of both the mother and the infant.

The objective of managing hypertension is to minimize the risk to the mother while ensuring the health of the fetus [8]. Methyldopa (CNS α agonist), Labetalol (combined α and β blocker), and Nifedipine (calcium‐channel blocker) are considered safe for use during pregnancy and are therefore recommended in clinical guidelines [3, 12]. Antihypertensive drugs can pass through the placenta. However, there are conflicting facts regarding the impact of these drugs on the mother and fetus during pregnancy [1, 13]. In a systematic review, medications such as Labetalol, other β‐blockers, Methyldopa, calcium channel blockers, and mixed/multi‐drug therapy were found to decrease the likelihood of severe hypertension by 30% to 70% when compared to a placebo or no treatment [14]. A Cochrane Library review study indicated that there is moderate‐certainty evidence suggesting that the use of antihypertensive drugs, as opposed to placebo or no antihypertensive drugs, can potentially reduce the risk of severe hypertension by half [15]. Results from the study by Salama et al. indicated that mothers in the control group (no medication) had a higher risk of developing severe hypertension, preeclampsia, renal impairment, ECG changes, placental abruption, and requiring repeated hospital admissions compared to those in the treatment groups (Methyldopa and Nifedipine). Furthermore, neonates born to mothers in the control group (no medication) were more likely to experience prematurity and require admission to the neonatal ICU compared to neonates born to mothers in the treatment groups [16].

On the other hand, a systematic review and meta‐analysis conducted by Bone et al. (2022) revealed that all frequently prescribed antihypertensive medications during pregnancy lower the likelihood of severe hypertension. However, Labetalol could also lower the incidence of proteinuria/preeclampsia and fetal/newborn mortality [14]. Ramzan et al. (2021) conducted a study that demonstrated that administering a 100mg oral dose of Labetalol three times daily to subjects significantly benefited most (87%) of patients in controlling maternal blood pressure. This treatment showed no adverse effects on the fetus [17]. In the randomized controlled trial study by Easterling et al. (2019), it was found that blood pressure control was significantly more common in women in the nifedipine group compared to those in the methyldopa group. However, there was no significant difference in blood pressure control between the nifedipine and labetalol groups or between the labetalol and methyldopa groups [12]. As a result, the conclusion about the clinical effectiveness of Labetalol, Nifedipine, and Methyldopa in treating HDP is controversial. To our knowledge, no study has evaluated the clinical outcomes and costs of Labetalol, Nifedipine, and Methyldopa in the treatment of HDP in Iran or worldwide. Therefore, this study aimed to compare the cost‐effectiveness of Labetalol versus Nifedipine and Methyldopa in treating HDP in Iran.

## 2 Materials and Methods

### 2.1 Study Design and Overview

In this model‐based economic evaluation, a decision tree model was developed to assess the cost‐effectiveness of three antihypertensive medications—labetalol, nifedipine, and methyldopa—for the management of hypertensive disorders during pregnancy in Iran. The analysis was conducted from the healthcare payer perspective over a 4‐month time horizon. This time horizon was considered appropriate because it covered the remaining duration of pregnancy from study enrollment (≥ 28 weeks of gestation) until delivery, thereby capturing all clinically relevant maternal outcomes associated with antihypertensive treatment. Within this framework, the cost‐effectiveness of labetalol was evaluated in comparison with nifedipine and methyldopa.

### 2.2 Population

The study population included in this economic evaluation was defined according to the baseline clinical characteristics of participants enrolled in the clinical trial conducted by Easterling et al. (ClinicalTrials.gov identifier: NCT01912677) [12]. Eligible participants were women aged 18 years or older, with a gestational age of ≥ 28 weeks, who had severe hypertension (defined as a systolic blood pressure ≥ 160 mmHg or a diastolic blood pressure ≥ 110 mmHg) requiring antihypertensive treatment.

The exclusion criteria were consistent with those of the original clinical trial and included women with significant medical comorbidities (e.g., asthma‐related complications, type 1 diabetes with microvascular complications, coronary artery disease, aortic dissection, or heart failure), those requiring an emergency cesarean section, those with known fetal anomalies, and those who had received antihypertensive medications within 12 h before enrollment [12].

### 2.3 Intervention and Comparator

The intervention consisted of oral labetalol at a dose of 200 mg administered once daily, as described in the clinical trial conducted by Easterling et al. Oral nifedipine (10 mg) and methyldopa (1000 mg as a single dose) were included as comparator interventions. These medications were selected because they were evaluated in the clinical trial and are commonly used for the management of hypertension during pregnancy [12].

### 2.4 Model Structure

**Figure 1:**
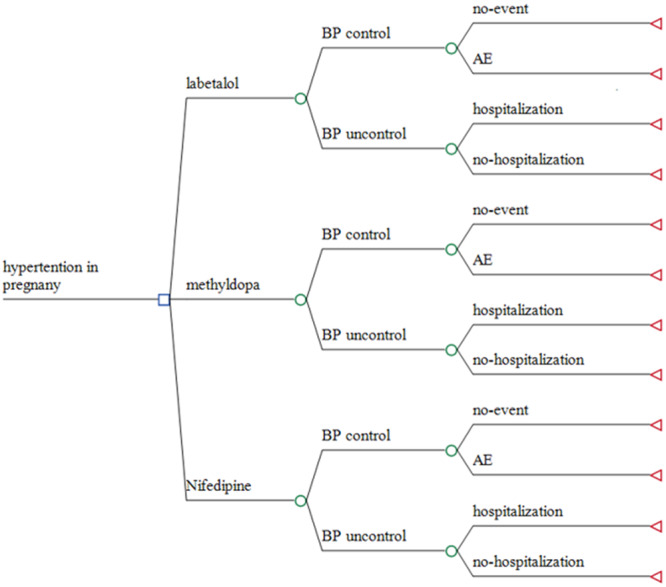
Decision tree model of health state.

A decision tree with a 4‐month time frame was created based on data from the Esterling et al. trial [12]. The decision tree model simulated the clinical pathways of pregnant women receiving one of the three treatment strategies, as illustrated in Figure 1. At the start of the simulation, all women were assumed to have hypertension and were subsequently classified according to whether their blood pressure was controlled or uncontrolled following treatment. In addition to treatment response, women were at risk of hospitalization and treatment‐related adverse events. Accordingly, the model incorporated the principal clinical pathways, including blood pressure control, uncontrolled hypertension, hospitalization, and treatment‐related adverse events. The clinical data were used to estimate the transition probabilities between health states in the decision tree model. Total costs and health outcomes, expressed as quality‐adjusted life years (QALYs), were estimated over the 4‐month time horizon. All costs were reported in US dollars (USD) after conversion from Iranian rials using an exchange rate of 64,529 Iranian rials per US dollar [18]. The cost‐effectiveness of the interventions was assessed using a willingness‐to‐pay (WTP) threshold of USD 18,261 per quality‐adjusted life year (QALY), based on the World Bank recommendations [19].

### 2.5 Efficacy

Treatment‐specific transition probabilities were estimated using the clinical outcomes reported in the trial conducted by Easterling et al. [12]. Where event rates, rather than probabilities, were reported, transition probabilities were estimated using the standard rate‐to‐probability conversion formula: (P = 1 ‐ e^{‐Rt}), where (P) represents the transition probability over the model cycle, (R) denotes the constant event rate, and (t) corresponds to the model cycle length (4 months).

### 2.6 Data Collection and Extraction Procedure

Model input parameters were identified using a stepwise approach. Data on clinical effectiveness were obtained primarily from the clinical trial conducted by Easterling et al., whereas data on adverse events, utility values, and costs were collected from multiple sources, including published studies, systematic reviews and meta‐analyses, and national databases.

A structured and reproducible approach was used to identify, extract, and standardize (where necessary) the model input parameters, including transition probabilities, utility values, and costs. The quality and relevance of all data sources were carefully assessed before inclusion in the model. Subsequently, the selected parameters were reviewed by an expert panel to ensure their consistency with current clinical practice and their applicability to the Iranian healthcare system.

### 2.7 Adverse Events

The probabilities of adverse events associated with labetalol and methyldopa were obtained from a published meta‐analysis, whereas those associated with nifedipine were derived from the clinical trial data [20, 21]. Only common, clinically relevant adverse events were included in the model.

### 2.8 Utility

Utility values were used to quantify health‐related quality of life (HRQoL) associated with the different health states included in the model. Baseline utility values for pregnant women were obtained from the study conducted by Lundsberg et al [22]. Due to the lack of published evidence on hypertension‐specific disutility among pregnant women, the disutility associated with hypertension was derived from studies conducted in the general population. Additionally, utility decrements associated with treatment‐related adverse events were obtained from previously published literature [23]. All utility inputs were carefully selected based on their relevance and the quality of the available evidence and were incorporated into the model to estimate quality‐adjusted life years (QALYs). A summary of the utility parameters and their corresponding data sources is presented in Table 1.

**Table 1:** Parameters for cost‐effectiveness analysis.

Reference	Distribution	Range	Value	Variables
**Probabilities**				[20]
BP control in the labetalol group BP control in the nifedipine group BP control in the methyldopa group Hospitalization	78 0.89 0.64 0.23	(0.9–0.66) (1–0.76) (0.74–0.54) (0.26–0.2)	Normal Normal Normal Normal	
**AE in labetalol group**				[20]
Drowsiness Headache Myalgia Nausea Vomiting Weakness	0.06 0.1 0.067 0.07 0.07 0.087	(0.07–0.05) (0.12–0.09) (0.08–0.06) (0.08–0.06) (0.1–0.07) (0.07–0.09)	Beta Beta Beta Beta Beta Beta	
**AE in the methyldopa group**				[20]
Drowsiness Headache Myalgia Nausea Vomiting Weakness	0.158 0.182 0.057 0.078 0.056 0.048	(0.13–0.18) (0.15–0.21) (0.05–0.07) (0.07–0.09) (0.05–0.06) (0.04–0.06)	Beta Beta Beta Beta Beta Beta	
**AE in nifedipine group**				[21]
Drowsiness Headache Hypotension Palpitation Vomiting	0.05 0.13 0.05 0.1 0.08	(0.04–0.06) (0.11–0.15) (0.04–0.06) (0.09–0.12) (0.07–0.09)	Beta Beta Beta Beta Beta	
**Cost**				[24, 25]
Nifedipine per 10 mg Methyldopa per 1000 mg Labetalol per 200 mg Admission to hospital Diagnosis test Additional medication AE management cost for Methyldopa or labetalol AE management cost for nifedipine	0.25 $ 0.24 $ 0.3 $ 4674 $ 232 $ 64 $ 703 $ 798 $	(0.2–0.3) (0.2–0.28) (0.26–0.35) (3973–5375) (197–267) (54–74) (598–808) (678–918)	Gamma Gamma Gamma Gamma Gamma Gamma Gamma Gamma	
**Utility**				[22, 23]
Utility of BP control The disutility of BP uncontrol Disutility of hospitalization The disutility of AE for labetalol or Methyldopa Disutility of AE for nifedipine	0.79 −0.025 −0.1 −0.195 −0.22	(0.67–0.91) −(0.02–0.03) −(0.09–0.12) −(0.17–0.22) −(0.19–0.25)	Beta Beta Beta Beta Beta	

### 2.9 Costs

Cost estimation was performed from the healthcare payer perspective and included direct medical costs associated with the management of hypertensive disorders during pregnancy. The components of care, including the type and frequency of healthcare services, were initially identified based on the NICE clinical guidelines [26]. To ensure the contextual relevance and validity of the model, an expert panel comprising seven obstetricians/gynecologists and cardiologists, each with at least 5 years of clinical experience, was convened. The panel reviewed and validated the clinical pathways, patterns of resource utilization, and cost components to ensure consistency with routine clinical practice in Iran. The analysis included direct medical costs associated with outpatient visits, medications, diagnostic tests, hospitalization, and the management of treatment‐related adverse events. Unit costs for healthcare services, including outpatient visits, diagnostic tests, and hospital care, were obtained from the official 2024 tariffs issued by the Iranian Ministry of Health. Drug acquisition costs were obtained from the national database of the Food and Drug Administration of Iran. All cost estimates were standardized and incorporated into the model for the economic evaluation.

### 2.10 Analyses and Outcomes

TreeAge Pro 2020 was used to develop the decision tree model for this study. The primary outcome was the incremental cost‐effectiveness ratio (ICER). The ICER was calculated as the difference in total costs divided by the difference in effectiveness between the two treatment options, measured in quality‐adjusted life years (QALYs). An intervention was considered cost‐effective if its ICER was below the willingness‐to‐pay (WTP) threshold of USD 18,261, based on World Bank estimates [19]. When ICERs were negative, they were interpreted in terms of dominance; a strategy that was both less costly and more effective was considered dominant. To account for uncertainty in the model results, both deterministic and probabilistic sensitivity analyses were performed, as described in Section 2.11. Reporting followed the CHEERS guidelines and relevant standards for health economic evaluations to ensure transparency, consistency, and appropriate reporting of the model structure, inputs, and outputs [27].

### 2.11 Sensitivity Analyses

To assess the robustness of the base‐case results to variations in input parameters, both one‐way sensitivity analysis and probabilistic sensitivity analysis (PSA) were conducted. For the one‐way sensitivity analysis, all probabilities, costs, and utility values (49 parameters) were varied within their respective standard errors (SEs). When standard errors were not available, probabilities and utility values were varied by ± 15%, and costs by ± 20%. The results were presented using a tornado diagram. For the PSA, the recommended probability distributions described by Briggs et al. were applied [28]. The beta distribution, which is bounded between 0 and 1, is suitable for modeling transition probabilities and utilities. Given its non‐negative support, the gamma distribution is appropriate for cost data. Based on these distributions, 10,000 Monte Carlo simulations were performed for the model parameters. A scatter plot of the simulated results was used to illustrate the cost‐effectiveness plane.

### 2.12 Ethical Considerations

The study protocol was approved by the Ethics Committee of Shiraz University of Medical Sciences (Approval Code: IR.SUMS.NUMIMG.REC.1404.022).

## 3 Results

### 3.1 Base Case Result

The total 4‐month cost in the labetalol group was lower than that in the nifedipine and methyldopa groups (USD 500 vs. USD 607 and USD 847, respectively), as shown in Table 2. In addition, women treated with labetalol achieved higher QALYs than those treated with methyldopa, but slightly lower QALYs than those treated with nifedipine (0.19 vs. 0.21 and 0.16, respectively). Accordingly, labetalol was dominant compared with methyldopa, as it was both less costly and more effective. The corresponding ICER was negative, indicating cost savings for labetalol relative to methyldopa in the treatment of pregnant women with hypertension. In the comparison between labetalol and nifedipine, the ICER was estimated at USD 5647 (95% CI: USD 4567–USD 7817) per QALY gained. Since this value is below the willingness‐to‐pay threshold, labetalol can be considered cost‐effective compared with nifedipine.

**Table 2:** Results of base‐case analysis.

Strategy	cost	Effect	Incr cost	Incr effect	ICER
Labetalol	500	0.19	—	—	
Methyldopa	847	0.16	240	0.04	−12,278
Nefidipine	607	0.21	107	0.02	5647

### 3.2 Sensitivity Analyses

**Figure 2:**
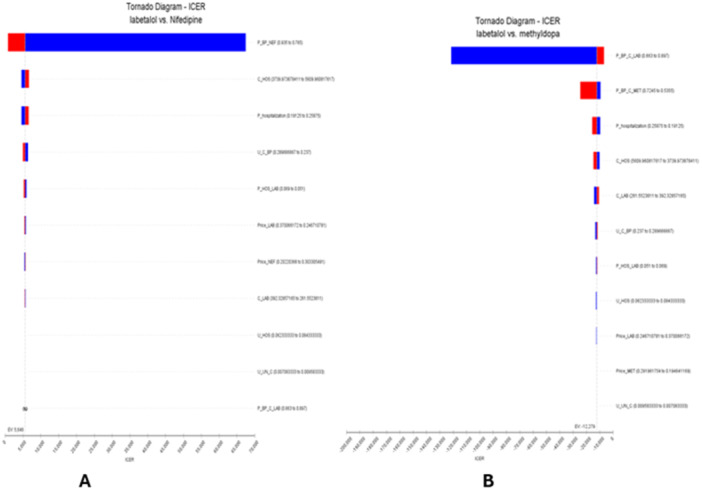
(A) Tornado diagram for the CEA analysis of Labetalol and Nifedipine. (B) Tornado diagram for the CEA analysis of Labetalol and Methyldopa.

When comparing labetalol with methyldopa, 48 parameters were varied within predefined ranges. All estimated ICERs remained negative; however, the results were most sensitive to the upper bound of the probability of blood pressure control in the labetalol group. In the comparison between labetalol and nifedipine, the tornado diagram similarly showed that the model was most sensitive to the upper bound of the probability of hypertension control. Nevertheless, these variations did not affect the overall conclusions, as the ICERs remained below the willingness‐to‐pay threshold. The results are presented in Figure 2A,B.

**Figure 3:**
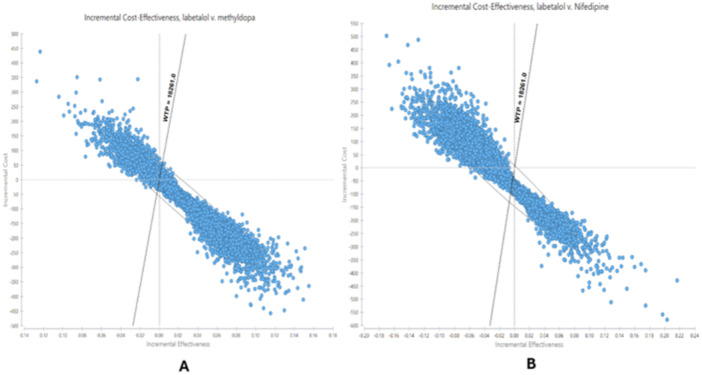
Scatter plot of 10,000 iterations of incremental cost and incremental QALY between (A) Labetalol treatment compared with Methyldopa, (B) Labetalol treatment compared with Nifedipine.

In the probabilistic sensitivity analysis (PSA) comparing labetalol and methyldopa, approximately 70% of the 10,000 iterations fell within the southeast (lower‐right) quadrant of the cost‐effectiveness plane, indicating that labetalol was both less costly and more effective in these simulations (Figure 3A).

In the comparison between labetalol and nifedipine, approximately 71% of the simulations fell above the willingness‐to‐pay threshold, indicating that the intervention was not cost‐effective in the majority of iterations across all WTP levels (Figure 3B).

## 4 Discussion

Hypertension and its related disorders are the most common conditions complicating pregnancy [29]. This was the first cost‐effectiveness study on patients with HDP in Iran and the world, which was conducted to investigate three medication strategies (Labetalol vs. Nifedipine and Methyldopa). The results showed that the effectiveness of Nifedipine, Labetalol, and Methyldopa in the treatment of HDP was 0.21, 0.19, and 0.16, respectively. It means that women with high blood pressure who were treated with Labetalol gained more QALYs than those treated with Methyldopa and fewer than those treated with Nifedipine.

In some studies, the effectiveness and safety of Nifedipine were compared with Labetalol or Methyldopa. A controlled trial conducted by Ainuddin et al. showed that oral Nifedipine achieved significantly earlier blood pressure control than oral Labetalol. Additionally, side effects such as bronchospasm, diarrhea, and constipation were considerably less frequent in the Nifedipine group than in those given Labetalol [21]. In the Easterling et al. randomized controlled trial, the administration of Nifedipine was associated with a higher rate of achieving blood pressure control compared to the use of Labetalol or Methyldopa [12]. The Shumard et al. study indicated that those who commenced treatment with Nifedipine experienced more rapid attainment of blood pressure control and had a lower frequency of requiring a second antihypertensive agent compared to those who began treatment with Labetalol [30]. In Beatty and Dangel's study, it was observed that patients who initiated treatment with oral Nifedipine during the postpartum phase of preeclampsia attained optimal blood pressure control in a notably reduced timeframe compared to those who began treatment with Labetalol, leading to a significantly shorter hospital stay [31]. In addition, Labetalol was compared with Methyldopa in patients with HDP. A meta‐analysis involving ten randomized controlled trials (RCTs) conducted by Patel et al. demonstrated that Labetalol offers more significant benefits than Methyldopa, particularly in achieving more effective and rapid blood pressure control.

Additionally, Labetalol is associated with a significantly lower incidence of maternal drowsiness as a side effect [20]. A study by Nasir showed that Labetalol regulates diastolic and systolic functions more rapidly and effectively than Methyldopa in treating gestational hypertension [13]. In this respect, our results aligned with these studies' results. However, these differ from the results of Sharma et al. and Maheshwari et al. [32, 33]. A recent large cohort study also demonstrated comparable maternal and fetal outcomes between labetalol and nifedipine [34]. A systematic review and meta‐analysis has also reported that there is no clear difference among oral antihypertensive agents in most maternal and fetal outcomes in pregnancy, although a slight preference for labetalol over nifedipine has been suggested for some outcomes [35].

On the other hand, the total cost of the Labetalol group ($500) was lower than that of the Nifedipine ($607) and Methyldopa group ($847). Considering the obtained negative ICER, treatment of HDP with Labetalol is a cost‐saving option compared with Methyldopa from the health system's perspective. This means that at a lower cost, Labetalol is more effective than Methyldopa in treating pregnant women with high blood pressure. However, the obtained ICER of $5647 per QALY is lower than the accepted threshold of $18,261 per QALY. The fact that the ICER is lower than the threshold shows that Labetalol is not cost‐effective compared to Nifedipine in treating HDP. Sensitivity analyses reinforce the robustness of these findings.

One of the reasons for the low cost of Labetalol treatment is due to the low rate of this drug compared to the other two drugs. On the other hand, it can be argued that Labetalol presents fewer fetal and neonatal adverse effects than the other two medications, resulting in the absence of newborn hospitalization associated with the reduction of mother hospitalization cost during Labetalol treatment when contrasted with the other two medicines. For instance, in a systematic review and meta‐analysis conducted by Bone et al., Labetalol was observed to be more effective and not only reduces the risk of proteinuria and preeclampsia but also decreases the likelihood of fetal or newborn death when compared to Methyldopa and calcium channel blockers [14]. In addition, the incidence of admissions to the intensive care unit was notably higher in the Nifedipine group, primarily due to a more significant proportion of low or very low birth weight infants compared to those born to mothers treated with Methyldopa or Labetalol [12]. Ramzan et al. and van de Vusse's study demonstrated that using Labetalol significantly improved the management of maternal blood pressure while simultaneously showing no adverse effects on fetal and neonatal health [3, 17]. However, Giannubilo et al. found that in the group treated with Labetalol, the rate of fetal worsening was higher, indicated by abnormal fetal heart tracing [36]. So far, the cost‐effectiveness study of these three drugs has not been done. But, according to Doshi et al., targeting a blood pressure of less than 140/90 mmHg, compared with 160/105 mmHg, in the treatment of chronic hypertension is a dominant, cost‐effective strategy that significantly reduces both neonatal and maternal mortality, while treating chronic hypertension at a lower threshold resulted in decreased costs of $600 million and increased effectiveness of 12,852 QALYs [37].

The primary goal of managing severe hypertension in pregnant individuals is to avert maternal cerebrovascular incidents and congestive heart failure while ensuring that cerebral perfusion and uteroplacental blood flow remain intact [29]. Based on the results of this study, Labetalol was cost‐effective compared to Methyldopa and not cost‐effective compared to Nifedipine. The Cochrane review concerning pharmacological treatments for hypertension in pregnancy indicated that the selection of antihypertensive drugs ought to be influenced by the clinician's experience, clinical judgment, and the specific circumstances of the patient, especially considering the known adverse effects until more definitive evidence is obtained [38]. The results of the review conducted by Amro et al. indicate that the choice of antihypertensive medication should be based on the potential adverse effects and the clinician's individual experience and familiarity with a particular medication. According to their findings, Labetalol is their first‐line agent, followed by calcium channel blockers [29]. Furthermore, the guidelines provided by the UK National Institute for Health and Care Excellence (NICE) concerning hypertension in pregnancy advocate for using Labetalol as the first‐line treatment, given its endorsement for administration to pregnant patients [39].

## 5 Limitation

This is the first cost‐effectiveness study conducted in Iran and among the first globally, evaluating three pharmacological strategies for hypertensive disorders of pregnancy (labetalol vs. nifedipine and methyldopa). A key limitation of the present study is the lack of high‐quality local clinical trials in Iran. As a result, effectiveness data were derived from clinical trials conducted in other countries.

## 6 Conclusions

This study showed that, over a 4‐month period in Iran, nifedipine was associated with the highest QALYs among the evaluated treatment options, followed by labetalol and methyldopa. Labetalol demonstrated a more favorable profile than methyldopa by providing higher QALYs at a lower cost, indicating its advantage as a preferred alternative to methyldopa. In comparison with nifedipine, labetalol involved a trade‐off between lower costs and lower QALYs. Findings from the probabilistic sensitivity analysis were consistent with the base‐case results, supporting the robustness of these conclusions. Overall, nifedipine and labetalol appear to be more favorable options than methyldopa for the management of hypertensive disorders of pregnancy in Iran.

## Author Contributions

**Zahra Goudarzi:** methodology, software, formal analysis, data curation, supervision, conceptualization, writing – original draft, visualization. **Mojtaba Nouhi:** conceptualization; methodology, writing – original draft, validation, data curation. **Najmeh Bordbar:** writing – original draft, investigation, conceptualization, methodology, writing – review and editing, supervision, project administration, data curation, resources.

## Funding

The authors have nothing to report.

## Ethics Statement

The study protocol was approved by the Ethics Committee of Shiraz University of Medical Sciences (Approval Code: IR.SUMS.NUMIMG.REC.1404.022). This study complied with the Declaration of Helsinki. Data analysis was performed on the records of patients who provided informed consent for using their data for research purposes.

## Conflicts of Interest

The authors declare no conflicts of interest.

## Transparency Statement

The lead author Najmeh Bordbar affirms that this manuscript is an honest, accurate, and transparent account of the study being reported; that no important aspects of the study have been omitted; and that any discrepancies from the study as planned (and, if relevant, registered) have been explained.

## Data Availability

The data that support the findings of this study are available on request from the corresponding author. The data are not publicly available due to privacy or ethical restrictions.
